# One visceral artery may be enough; successful pancreatectomy in a patient with total occlusion of the celiac and superior mesenteric arteries

**DOI:** 10.1186/s12893-018-0352-0

**Published:** 2018-05-16

**Authors:** Evangelos Tagkalos, Florian Jungmann, Hauke Lang, Stefan Heinrich

**Affiliations:** 1 Department of General, Visceral and Transplantation Surgery, Johannes Gutenberg University Hospital, Langenbeckstrasse 1, 55131 Mainz, Germany; 2 Department of Diagnostic and Interventional Radiology, Johannes Gutenberg University Hospital, Mainz, Germany

**Keywords:** Pancreaticoduodenectomy, Mesenteric arteries, Occlusion, Celiac trunk

## Abstract

**Background:**

The anatomic variations of the visceral arteries are not uncommon. The liver arterial blood supply shows 50% variability between humans, with the most common anatomy being one hepatic artery arising from the celiac trunk and one pancreatico-duodenal arcade between the celiac trunk and the superior mesenteric artery. Occlusion of one artery are mostly asymptomatic but may become clinically relevant when surgery of the liver, bile duct or the pancreas is required. If these pathologies are not reversible, an oncologic pancreatic head resection cannot be performed.

**Case presentation:**

We report the case of a 64-year-old Caucasian female patient with a locally advanced, resectable adenocarcinoma of the pancreas with complete atherosclerotic occlusion of the celiac trunk and the superior mesenteric artery. This vascular anomaly was missed on the preoperative imaging and became known postoperatively. A collateral circulation from a hypertrophic inferior mesenteric artery to the celiac trunk and the superior mesenteric artery compensated the blood supply to the visceral organs. The postoperative course was complicated by an elevation of the transaminases AST/ALT, which normalized under conservative treatment with alprostadil (prostavasin™) and anticoagulation, since angiographic recanalization failed. The patient recovered fully and was discharged at the 14th postoperative day. Two years later, she required endovascular repair of an aortic rupture during which the inferior mesenteric artery was preserved.

**Conclusion:**

This case underlines the natural potential of the human body to adapt to chronic arterial malperfusion by creating a collateral circulation and supports the need for adequate preoperative imaging, including a proper arterial phase before upper abdominal surgery.

## Background

The blood supply of the liver is variable and only 50% of humans present with a standard vascular anatomy of one hepatic artery arising from the celiac trunk and a pancreatico-duodenal arcade between the hepatic and the superior mesenteric arteries (SMA) [1]. Chronic occlusion of one artery exists in approximately 10–20% of patients, but can often be compensated and therefore usually remains asymptomatic due to the dual blood supply [2–5]. The most frequent compensation is the hypertrophy of the pancreatico-duodenal arcade [6].

Asymptomatic occlusion may become clinically relevant when surgery of the liver, bile duct or the pancreas is required; the oncologic resection of the pancreatic head (e.g. Whipple procedure) requires the ligation of the gastroduodenal (GDA), and the pancreatico-duodenal arteries (PDA). Gastrointestinal and biliary anastomoses require adequate arterial flow. Therefore, a celiac trunk stenosis is critical and a complete occlusion is considered a contraindication for many of these procedures.

We report the case of a patient with a locally advanced, resectable adenocarcinoma of the pancreas with complete atherosclerotic occlusion of the celiac trunk and the SMA.

## Case presentation

**Fig. 1:**
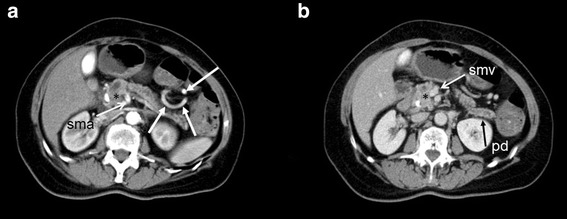
Transverse CT images preoperatively with arterial (**a**) and venous (**b**) phases: the pancreatic duct (pd) is dilated, a stent had been implanted into the bile duct due to biliary obstruction of the tumor in the pancreatic head (*). The arterial collaterals are visible in the left upper abdomen (arrows) (sma): superior mesenteric artery, smv: superior mesenteric vein)

A 64-year-old female patient suspicious of cancer of the pancreatic head with a recent history of acute pancreatitis and cholangitis was admitted to our department for further treatment in April 2012. The patient had had coronary artery bypass surgery, diabetes and arterial hypertension. Laboratory findings revealed cholestasis, and endoscopic retrograde cholangiopancreatography (ERCP) showed a stenosis in the distal bile duct which was highly suspicious of a pancreatic malignancy. Computed tomography (CT) scan and magnetic resonance imaging (MRI) images, 2 months and 3 weeks prior to surgery, were demonstrated by a radiologist in the preoperative conference: a double duct sign, but no signs of metastases were found, and the tumor had contact to the superior mesenteric artery without suspect of a vascular invasion (Fig. 1).

The patient underwent a classic Whipple procedure (partial duodenopancreatectomy) with regional lymphadenectomy in May 2012. Intraoperatively, the arterial pulse in the liver hilum appeared weak, but flow increased after test clamping of the GDA. Therefore, resection was completed. The dissection of the mesopancreas was performed on the SMA. The pancreatico-jejunostomy was performed according to Warren & Kartell using 4–0 PDS and 6–0 PDS sutures for the duct-to-duct anastomosis. About 15 cm distal to this anastomosis, the hepatico-jejunostomy was done with 5–0 PDS interrupted sutures. The intestinal passage was reconstructed by a gastroenterostomy (3–0 PDS) and a Braun anastomosis using 4–0 PDS.

### Postoperative course

**Fig. 2:**
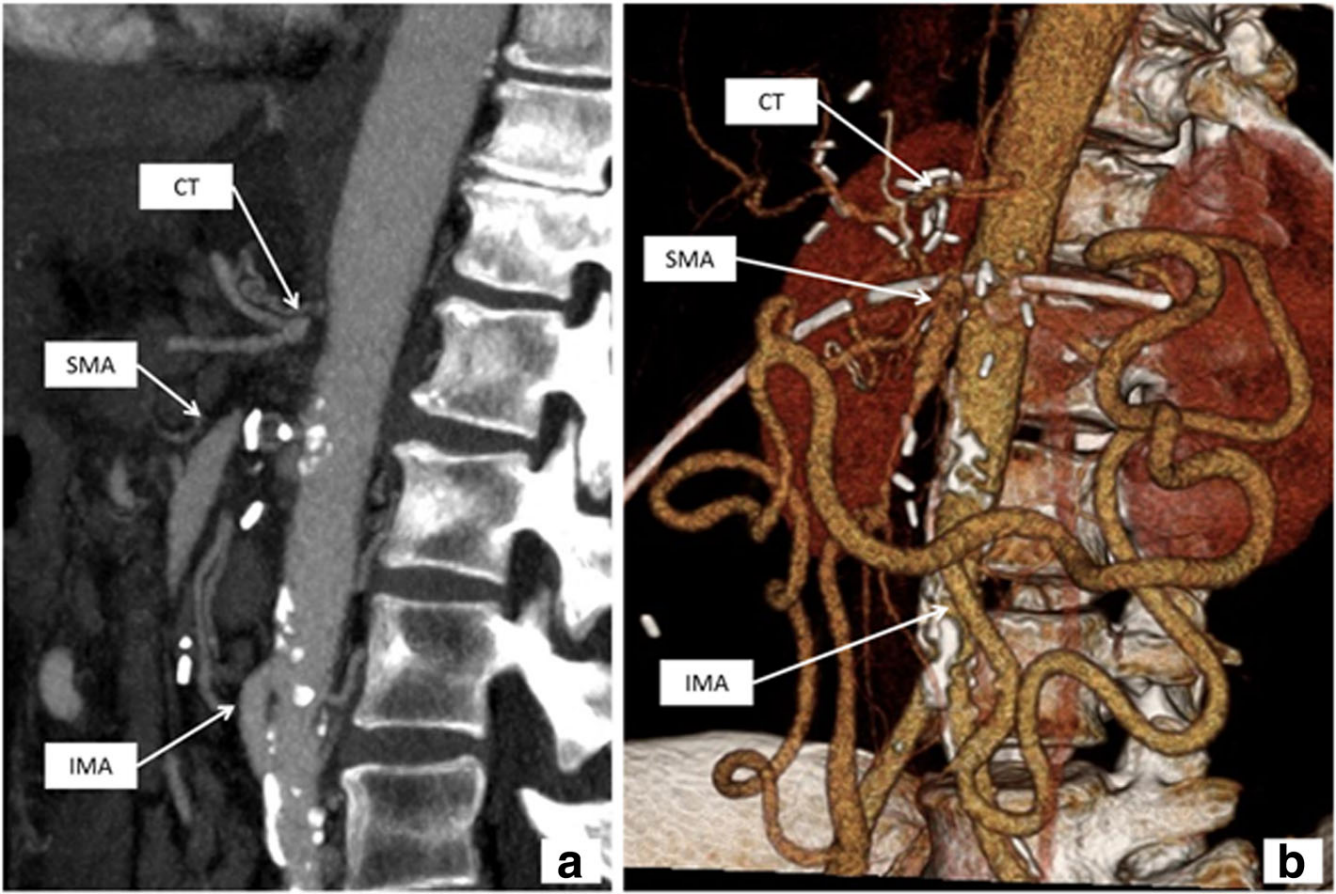
Sagittal reconstruction of a computed tomography scan (**a**) demonstrates proximal occlusion of celiac trunk (CT) and SMA with poststenotic dilatation of the SMA. Note the hypertrophic IM. Volume rendering (**b**) shows collateral arteries from IMA to SMA (hypertrophic anastomosis of Riolan)

**Fig. 3:**
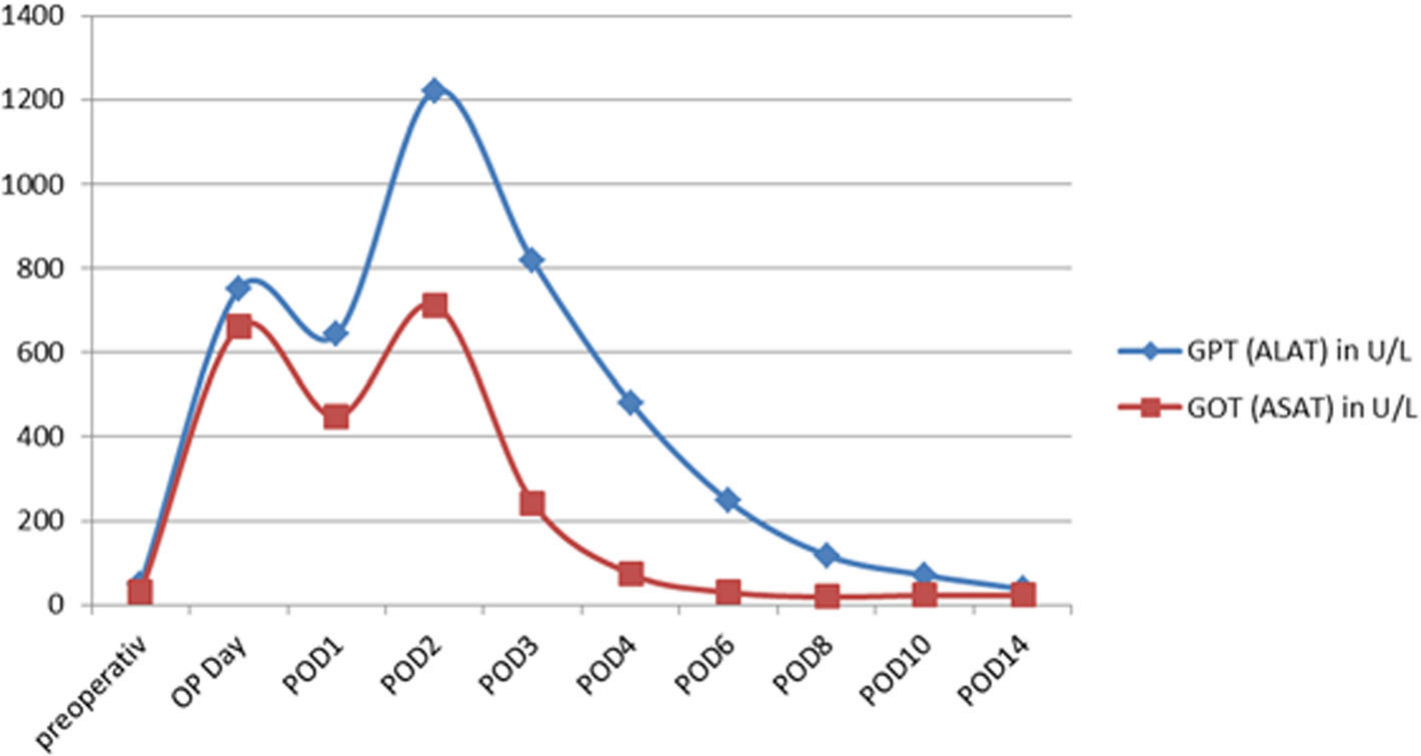
Postoperative course of transaminases AST/ALT

Postoperative transaminases were elevated and peaked on postoperative day (POD) 3 (AST 713 U/L, ALT 1222 U/L). Therefore, abdominal CT with intravenous contrast was performed and revealed a chronic occlusion of both the celiac trunk and the SMA. A large-caliber inferior mesenteric artery (IMA) had strong collaterals to the SMA and celiac trunk (Fig. 2). Emergency angiography confirmed chronic occlusion of the SMA and celiac trunk without any possibility for interventional therapy. In the absence of treatment alternatives, continuous alprostadil (prostavasin™) infusion and anticoagulation with unfractionated heparin were initiated and continued over 7 days. During this treatment, transaminases decreased continuously and remained normal thereafter (Fig. 3).

### Histopathological findings

The final histology revealed a 3.2 cm poorly differentiated adenocarcinoma of the pancreatic head with infiltration of the distal bile duct and the peripancreatic tissue. Moreover, the tumor had spread to 4/18 lymph nodes, and perineural and vascular infiltration were detected (pT3, pN1 (4/18), M0, G3, V1, Pn1, R0).

### Outcome

No further complications occurred in the postoperative course. In particular, no signs of intestinal hypoperfusion or anastomotic leak occurred. The patient was discharged 2 weeks after the operation in a very good condition.

### Follow-up

**Fig. 4:**
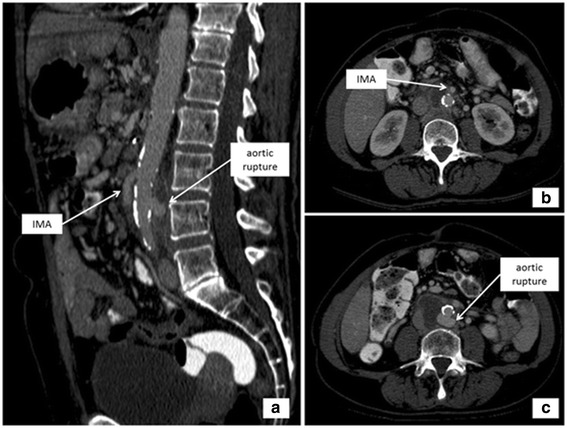
Sagittal reconstruction of computed tomography scan (**a**) and axial images (**b**, **c**) revealed abdominal aortic pseudoaneurysm at the dorsal circumference of the aorta below the ostium of the IMA due to tumor relapse

The patient received 6-months of adjuvant gemcitabine chemotherapy and presented in excellent general condition for a follow-up 12 months after surgery. Two years after surgery, the patient required emergency endovascular treatment (EVAR) of an aortic rupture. The aortic rupture extended from the aortic bifurcation to the renal arteries. At that time, also tumor recurrence was found (Fig. 4). The IMA was spared during stent placement in order to preserve intestinal perfusion. After this intervention, the patient received 5 cycles of palliative gemcitabine chemotherapy and later changed to FOLFOX4 in December 2014 due to tumor progression. The patient died 34 months after the pancreas resection.

## Discussion and conclusions

Splanchnic vascular stenosis or occlusion can be caused by a variety of diseases. One of the most frequent causes of a stenosis of the celiac trunk is a hypertrophic arcuate ligament resulting in an extrinsic obstruction of the artery [3, 7, 8], which can usually be solved intraoperatively by dissecting the ligament [9]. Angiographic studies revealed asymptomatic severe stenosis of celiac or superior mesenteric arteries in up to 50% of patients with atherosclerosis, and synchronous severe stenosis of both arteries are present in up to 5% of these patients [7, 10]. A percutaneous transluminal stent placement or even a vascular bypass surgery may be performed to improve the perfusion of the celiac trunk. If these interventions are not feasible, a Whipple procedure is considered impossible. In contrast, the hypertrophic pancreatico-gastroduodenal arcade may be preserved during distal pancreas resection [11, 12].

In this case, the arterial collaterals as consequence of the arterial occlusion were misdiagnosed as venous collaterals during the preoperative conference. Upon postoperative CT scan, the late arterial phase clearly demonstrated the occlusion of both vessels. Due to the chronicity of the arterial occlusion, hypertrophic collaterals, e.g. through the anastomosis of Riolan, had been formed and prevented severe complications. In contrast, the resection of the celiac trunk or the SMA without development of collaterals may have fatal consequences.

In summary, the synchronous chronic occlusion of the celiac trunk and the SMA, as reported in this case, is extremely rare, while cases of occlusion of one of both is well known in the literature. If this arterial situation would have been detected preoperatively, a bypass procedure would have been performed during pancreas resection in order to prevent serious complications, or surgery would have been denied. Although our patient tolerated major pancreas surgery well, procedures impairing the IMA (i.e. left hemicolectomy) could have been lethal for her. Since this arterial situation was well documented, the IMA was preserved during the emergency treatment of an aortic rupture 24 months postoperatively.

Although chronic arterial occlusion can be compensated through collateralization, cases as the above emphasize the importance of proper arterial imaging prior to operations of the upper abdomen. Moreover, careful assessment of the available imaging by trained radiologists is mandatory.

## Abbreviations

CT: Computed tomography
ERCP: Endoscopic retrograde cholangiopancreatography
EVAR: Endovascular aneurysm repair
GDA: Gastroduodenal artery
IMA: Inferior mesenteric artery
MRI: Magnetic resonance imaging
PDA: Pancreatico-duodenal artery
POD: Postoperative day
SMA: Superior mesenteric artery
SMV: Superior mesenteric vein
